# Diphtheria and Antitoxin

**Published:** 1895-02

**Authors:** J. Kennedy Corss

**Affiliations:** Norcross Building; Atlanta


### *CORRESPONDENCE.*

---

### DIPHTHERIA AND ANTITOXIN.

ATLANTA, January 15, 1895.

*Atlanta Medical and Surgical Journal:*

Bacteriology has added much to our knowledge of diphtheria, and aided both in the prevention and treatment of the disease.

The serum therapy, or antitoxin treatment, may result in a great decrease in the death-rate, and if immunity by inoculation of healthy persons proves a success, it may prevent the terrible epidemics which have occurred in the past. As yet the entire serum treatment is purely experimental, and may do positive harm by taking the place of older and tried remedies until it is too late for them to be used with benefit to the patient.

It is right that a treatment which seems rational and promises such wonderful improvement over any previous methods employed in the dread disease should receive the unanimous encouragement of the profession. At the same time we owe a duty to each case we treat, whether it be in private practice or hospital service; and as long as there is no antagonism between the use of antitoxin and the remedies previously employed, it is the duty of the physician to follow the methods approved by past experience, whether the serum be used or not. In the light of statistics of cases treated by antitoxin, and these are always presented as favorably as possible, no one can as yet claim it to be a specific. The number of deaths, complications, and cases requiring tracheotomy does not warrant one using the serum to the exclusion of other remedies until it is far better established in its true position in medicine than at the present time.

Before calling attention to the treatment of a complication which occurred in one of my cases, I wish to give a few statistics from the new treatment, showing the number of cases requiring tracheotomy.

In a series of eighty cases in the Eastern Hospital, London, sixty-one showed the bacilli of diphtheria, and of these tracheotomy was required in nine cases, with only three deaths—a remarkably low mortality for tracheotomy. In the Royal Hospital for Children, Aberdeen, six cases were treated with antitoxin, and of these five required tracheotomy, with three deaths. In St. Bartholomew's Hospital, out of eighteen cases under antitoxin treatment, ten required tracheotomy, with only three deaths.

These cases, taken from the hospitals where the treatment could be carefully carried out, must show that the disease is far from being conquered, and the physician must not neglect any therapeutic indication and be prepared to operate at any moment.

In one case which came under my care and tracheotomy became necessary, there occurred one of the most distressing complications, and one which is often fatal. A. H., female, age five years, was attacked with diphtheria on March 3, 1893. Treatment was with bichloride of mercury, tincture of the chloride of iron, and stimulants, in addition to local applications. It was a stubborn case, and two days later tracheotomy became necessary to relieve the dyspnœa. Membrane formed below the tube, and on the second night there was complete obstruction of the right bronchus, entirely preventing the entrance of air. The obstruction was beyond reach of tracheal forceps and death seemed imminent, while the struggle for air was most painful to watch. I at once gave hypodermic of strychninæ sulph., gr. $\frac{1}{30}$ in a f℥ ss. of whisky. To remove the obstruction, used a preparation of glycerinum pepticum (Fairchild's) f℥ i., with pure hydrochloric acid gtt. iv., in a f℥ i. of water, at about 115° F. By attaching a rubber catheter to the end of an ordinary hand atomizer, first removing the tip and attaching it to the other end of the catheter, it was possible, by placing patient on right side, to reach the opening of the bronchus through the tracheotomy tube. In this way I directed a fine spray of the pepsin solution directly upon the obstructing membrane. Dyspnœa at first increased from the swelling of the plug, and I did artificial respiration. In about ten minutes there was slight movement of the right chest, and a cough brought up a partly digested membranous cast of the bronchus, which was easily removed with the tracheal forceps. The relief was wonderful. On each of the two succeeding nights the obstruction recurred in the same position, and was relieved by the same treatment.

The case seemed to improve for nine days, and then died from heart failure. I do not know that this method would give relief in all cases, but it is certainly worth trying, and is very simple and easy to prepare. The chief objection to it is the danger of pneumonia from the introduction of a foreign substance into the lung; but that is not to be considered where the immediate danger is so great, and the very presence of the obstructing membrane will likely prevent its entrance below the bronchus. When diphtheritic membrane forms to any extent below the tracheomoty tube, the case is generally fatal, and anything which will relieve the suffering of the patient, as in this case, and tend to remove the cause, is at least worth the trial. J. KENNEDY CORSS, M.D.

*Norcross Building.*

